# Influence of the Deposition Parameters on the Properties of TiO_2_ Thin Films on Spherical Substrates

**DOI:** 10.3390/ma16144899

**Published:** 2023-07-08

**Authors:** Maria Covei, Cristina Bogatu, Silvioara Gheorghita, Anca Duta, Hermine Stroescu, Madalina Nicolescu, Jose Maria Calderon-Moreno, Irina Atkinson, Veronica Bratan, Mariuca Gartner

**Affiliations:** 1 Department of Product Design, Mechatronics and Environment, Transilvania University of Brasov, 29 Eroilor Bd., 500036 Brasov, Romania; maria.covei@unitbv.ro (M.C.);; 2 “Ilie Murgulescu” Institute of Physical Chemistry, Romanian Academy, 202 Splaiul Independentei St., 060021 Bucharest, Romania

**Keywords:** TiO_2_ thin film, photocatalysis, glass bead substrate, sol-gel, dilution ratio, number of layers, morphology, structure, stability

## Abstract

Wastewater treatment targeting reuse may limit water scarcity. Photocatalysis is an advanced oxidation process that may be employed in the removal of traces of organic pollutants, where the material choice is important. Titanium dioxide (TiO_2_) is a highly efficient photocatalyst with good aqueous stability. TiO_2_ powder has a high surface area, thus allowing good pollutant adsorption, but it is difficult to filter for reuse. Thin films have a significantly lower surface area but are easier to regenerate and reuse. In this paper, we report on obtaining sol-gel TiO_2_ thin films on spherical beads (2 mm diameter) with high surface area and easy recovery from wastewater. The complex influence of the substrate morphology (etched up to 48 h in concentrated H_2_SO_4_), of the sol dilution with ethanol (1:0 or 1:1), and the number of layers (1 or 2) on the structure, morphology, chemical composition, and photocatalytic performance of the TiO_2_ thin films is investigated. Etching the substrate for 2 h in H_2_SO_4_ leads to uniform, smooth surfaces on which crystalline, homogeneous TiO_2_ thin films are grown. Films deposited using an undiluted sol are stable in water, with some surface reorganization of the TiO_2_ aggregates occurring, while the films obtained using diluted sol are partially washed out. By increasing the film thickness through the deposition of a second layer, the roughness increases (from ~50 nm to ~100 nm), but this increase is not high enough to promote higher adsorption or overall photocatalytic efficiency in methylene blue photodegradation (both about 40% after 8 h of UV-Vis irradiation at 55 W/m^2^). The most promising thin film, deposited on spherical bead substrates (etched for 2 h in H_2_SO_4_) using the undiluted sol, with one layer, is highly crystalline, uniform, water-stable, and proves to have good photocatalytic activity.

## 1. Introduction

Due to water scarcity in many parts of the world, wastewater treatment (WWT) targeting its reuse for different applications (e.g., in agriculture, industry, domestic activities, etc.) represents an important aspect of sustainable communities [1,2].

However, there are many organic pollutants (dyes, pesticides, phenol, antibiotics) that cannot be (fully) removed using traditional methods. For the treatment of wastewater loaded with low concentrations of these pollutants (in the ppm range), the use of advanced oxidation processes, such as photocatalysis, may be a viable option [3,4]. However, its applications are mostly reported at the laboratory or pilot plant scale [4]. 

For up-scaled operation, the associated process cost needs to be reduced by using (a) non-toxic, low-cost, easily recoverable (through filtration) photocatalysts, (b) natural solar-/Vis-radiation, and (c) up-scalable technologies from laboratory to demonstrator level and beyond, when integrated into WWT plants [4,5,6].

One frequent discussion on the selection of the appropriate photocatalysts is the choice between powder and thin film. Powders have a higher specific surface area and more adsorption sites available for the pollutant molecules, thus being more efficient than thin films; however, their use in suspension reactors is limited by the particles’ agglomeration, by the radiation losses (due to scattering) and the limited powder recovery. They can also cause secondary pollution as there is a risk of nanoparticle release in the environment [7]. The use of thin film-photocatalysts can solve these problems, but these types of materials are generally less efficient in pollutant removal as they are characterized by lower specific surface area compared to powders [7,8,9]. Loading thin photocatalytic films on spherical bead substrates made of glass, silica, aluminum, ceramics, chitosan, alginate, etc., was recently reported as a viable path to prepare efficient and stable photocatalysts with large specific surface areas that are easy to filter, treat and reuse [7,9,10,11,12,13,14,15,16,17,18,19]. Thus, the use of photocatalytic beads in the reactor is much more attractive compared to powders.

In the photocatalytic bead development, titanium dioxide (TiO_2_) is intensively investigated [7,9,10,11,12,13,14,15,17,18,19,20] as it has been the main photocatalyst choice for several decades due to its high photocatalytic efficiency and its good aqueous stability over a broad pH range [21,22]. Photocatalytic beads obtained by coating different spherical substrates (glass, silica, alumina, etc.) with a thin film based on commercial titanium dioxide (Degussa P25, Aeroxide 25) or obtained by various techniques such as the sol-gel method, fluidized bed chemical vapor deposition, etc., were successfully employed in the degradation of different organic pollutants (dyes, pesticides, phenols) under UV irradiation [10,11,12,13,14,15,16,17,18,19]. In these studies, TiO_2_ synthesis mainly followed the sol-gel route, and the immersion/dip coating method was further involved in coating the beads. These are versatile, cost-effective techniques (low processing temperature, low-cost equipment) that allow deposition on substrates with different geometry and shapes and allow good control of the photocatalyst properties (particles with good homogeneity, controllable morphology, and crystallinity when an appropriate annealing treatment is applied) [5,11].

The degradation of different dyes such as methylene blue [12], methyl orange [11,14], or reactive red, acid brown [15,17] is reported on TiO_2_-coated beads. Thus, TiO_2_ coatings were deposited on various bead substrates (glass, alumina, silica) with a thickness of ~35 nm; the results showed that the morphology and the efficiency in the standard methylene blue removal are influenced by the substrate type and the deposition conditions, and the best results correspond to the TiO_2_ coated on silica beads [12].

Porous glass beads coated with a thin film of TiO_2_ showed good efficiency in the methyl orange degradation under UV irradiation: 65% up to entire discoloration of the methyl orange after 30 min of irradiation depending on the Ti (at. %) content in the coating and the catalyst load [11]. Sol-gel S, N co-doped TiO_2_ nanoparticles immobilized on glass beads using the dip coating technique, tested in the methyl orange photodegradation in both laboratory- and in large-scale photoreactors exhibited up to 95% removal efficiency under solar irradiation for 2 h [14].

The photocatalytic efficiency of TiO_2_ coated beads using different substrates (glass, silica, clay) in the degradation of phenol, different types of pesticides, or emerging contaminates are reported both at laboratory and pilot plat scale [10,13,18,19,20]; the removal efficiencies reach values of 70–92% for the investigated pesticides under UV irradiation [10,13,18], while 15 emerging contaminants were successfully degraded to a few μg/L (removal efficiency 70–100% after 60 min of irradiation) by solar photocatalysis in compound parabolic collector (CPC) photoreactors. Moreover, the photoactivity of the investigated TiO_2_-coated glass beads is preserved even after five testing cycles [20].

Besides the good photocatalytic efficiency in pollutant(s) removal, the photocatalytic beads’ stability in the aqueous environment under irradiation is essential. This is correlated with a good adherence of the coating to the spherical substrate, which can be improved by etching the spherical beads (glass, silica) in concentrated acid solutions (nitric acid, sulphuric acid, hydrochloric acid or hydrofluoric acid) [14,17,19] when the bead porosity and specific surface area are expected to increase.

In this study, we report on photocatalytic beads obtained by coating glass spherical substrates with sol-gel TiO_2_ aimed at the degradation of the standard methylene blue (MB) pollutant (ISO 10678:2010 [23]) from diluted solution (10 ppm) under UV-Vis irradiation at low irradiance value. Methylene blue is frequently used as a target molecule for degradation in the photocatalytic process, mainly due to the simplicity in the assessment of its degradation efficiency that is based on UV-Vis spectrometry. Methylene blue is a dye with a high molar absorbance that allows the UV-Vis detection of the color change from blue up to colorless during photobleaching supported by the photocatalyst in the aqueous medium under irradiation [23,24].

The influence of the substrate morphology (correlated with the duration of the etching treatment) of the sol dilution with ethanol (1:0 or 1:1) and the number of deposited layers (1 or 2) on the structure, morphology, chemical composition, photocatalytic performance and stability of the TiO_2_ coating was investigated; based on these results, the best TiO_2_ photocatalytic beads in the experimental conditions were outlined.

## 2. Materials and Methods

Thin TiO_2_ films obtained following the sol-gel method were deposited on glass beads with a diameter of 2 mm. Before deposition, the substrates were etched in sulfuric acid (Scharlau, 96%) for 0.5 h up to 48 h to remove possible impurities as well as to increase the surface area and activate it. This treatment was followed by washing with water, detergent, and, finally, ethanol (15 min ultra-sonication for each). The impact of the etching duration on the substrate morphology and chemical composition was investigated to select the optimum etching duration.

**Figure 1:**
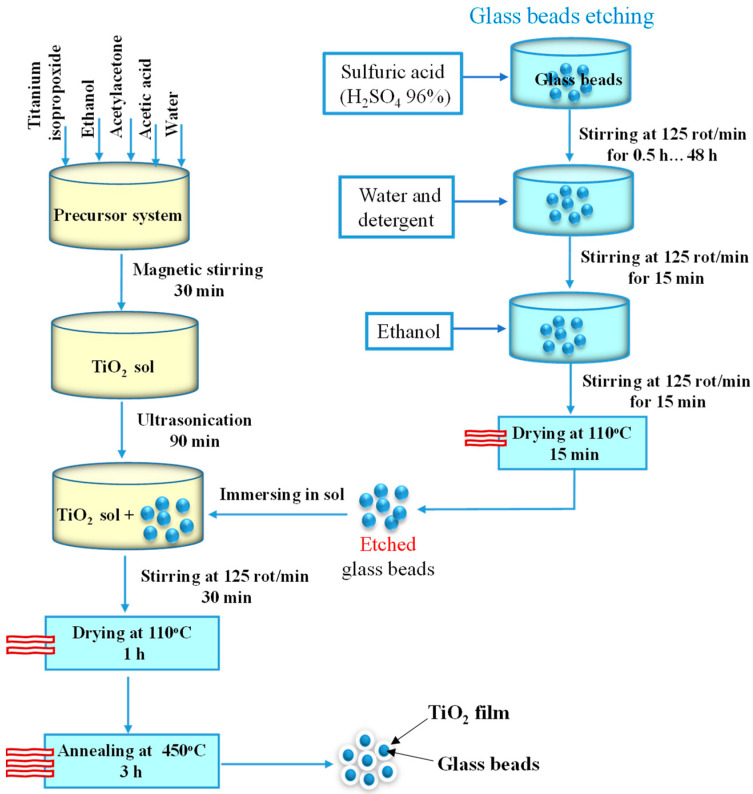
Diagram of the thin film deposition methodology.

The sol for the TiO_2_ layer was obtained by mixing titanium isopropoxide (Aldrich, >97%), ethanol (Chimreactiv, 99.5%), acetylacetone (Scharlau, 99%), acetic acid (Scharlau, 99.8%), and water in a volumetric ratio of 20:16:0.89:0.18:2.4, for 30 min under magnetic stirring, followed by 90 min ultrasonication. The previously etched substrate (1 g of beads) was immersed in the sol (5 mL) and stirred for 30 min, using an orbital stirrer, followed by drying (110 °C—1 h) and annealing (450 °C—3 h) in a furnace Nabertherm B150. The methodology of the thin film deposition is schematically represented in Figure 1.

The crystallinity of the thin films was investigated using a Bruker D8 Discover X-ray Diffractometer (CuKα1 = 1.5406Å, step size 0.025, scan speed 1.5 s/step, 2θ from 5 to 80°). Scanning electron microscopy (SEM) was done on a Hitachi SEM S-3400 N type 121 II apparatus coupled with a Thermo Scientific (Waltham, MA, USA) UltraDry energy dispersive X-ray spectrometer (EDX). Surface topology was analyzed through atomic force microscopy (AFM) using an NT-MDT microscope, model NTGRA PRIMA EC, working in semicontact mode with Si-tips, NSG10, force constant 0.15 N/m, tip radius 10 nm. Roughness was estimated using the AFM software (Nova1138) on a 20 μm × 20 μm surface.

The DR UV-Vis spectra were recorded using a Perkin Elmer Lambda (Shelton, CT, USA) 35 spectrophotometer equipped with an integrating sphere. The measurements were carried out in the 900–200 nm wavelength range using spectralon as a reference and a special holder. The reflectance measurements were converted into absorbance spectra using the Kubelka–Munk function. The optical band gap was calculated using the Tauc plot, which assumes that the absorption coefficient, α, is related to the band gap energy of the semiconductor by applying Equation (1):

(1) (α∗hν)1n=B∗(hν−Eg)

where h is the Planck constant, ν is the photon frequency, and B is a constant. The factor n has different values, depending on the nature of the electronic transitions, and can be ½ or 2 for the direct or indirect transition, respectively. The band gap is usually measured from diffuse reflectance spectra, by replacing α with F(R). E_g_ is determined from the x axis intercept of the graphical representation of (F(R) ∗ hν)^1/n^ vs. hν, for *n* = 2 [25].

Souri et al. [26] showed that the η value can be determined using the absorption spectra from the slope of the linear part of the graphs of ln[A(λ)/λ] versus 1/λ to determine the optical bandgap without any presumption of the nature of the transition by the absorption spectra [26]. Elemental analysis (XRF) of the films was performed in a vacuum using a Rigaku ZSX Primus II spectrometer (Tokyo, Japan). The results were analyzed using EZ-scan combined with Rigaku SQX (version 5.18) fundamental parameters software capable of automatically correcting all matrix effects, including line overlaps.

Contact angle analysis was performed using OCA20 equipment, with water as the testing liquid (5 μL droplet). Due to the small area of the beads, the contact angle measurements could not be performed directly on the beads and were done on glass slides (2 × 2 cm^2^) purchased from Citoglas and etched in 98% sulfuric acid, similar to the glass beads for 0.5 up to 48 h.

A circular photo-reactor with two UV light sources (UVA, 340–400 nm, λ_UV,max_ = 365 nm, Philips, Amsterdam, The Netherlands) and five VIS light sources (TL-D Super 80 18W/865, 400–700 nm, λ_VIS,max_ = 565 nm, Philips), with a total average irradiance of G = 55 W/m^2^ was used. The UV share in the radiation is 10%, to resemble solar radiation conditions at a much lower irradiance value. The irradiance value was recorded using a Kipp and Zonnen pyranometer.

The photocatalytic experiments used the standard methylene blue (MB, 99.8%, Merck) pollutant at the initial concentration of 10 ppm (ISO 10678:2010 standard determination of photocatalytic activity of surfaces in an aqueous medium by degradation of methylene blue) [16].

The photocatalytic beads (1 g) were immersed in a 20 mL aqueous pollutant solution. Before irradiation, 1 h contact between these was allowed in the dark to reach the adsorption/desorption equilibrium. The photodegradation efficiency, *η*, was calculated based on the initial absorbance of the pollutant solution (*A*_0_) and the absorbance recorded hourly up to 8 h of irradiation (*A*) at the maximum absorbance wavelength of the pollutant (λ_MB_ = 664 nm) using a UV-Vis-NIR spectrophotometer (Perkin Elmer Lambda 950) and applying Equation (2):

(2) $$\eta =\frac{A_{0}-A}{A_{0}}\times 100$$

To assess the contribution of the adsorption to the entire removal efficiency, experiments were also conducted in the dark using the same set-up and conditions as in the photocatalytic experiments but in the dark, measuring the solution absorbance hourly during 1–9 h, corresponding to contact times equal to those used in the photocatalytic experiments.

## 3. Results and Discussion

The influence of the parameters of interest on the photocatalyst TiO_2_ thin films deposited on glass beads was investigated, considering: (a) the substrate etching duration, (b) the sol dilution with ethanol, and (c) the number of deposited layers.

### 3.1. Influence of the Etching Duration on the Substrate Properties

Surface structure and morphology can significantly influence thin film growth. In order to increase the specific surface area and create more nucleation sites on the substrate, etching in H_2_SO_4_ was proposed. The etching duration was varied to create a large specific surface without damage from concentrated H_2_SO_4_. It is also expected that a longer etching duration will lead to the formation of micro- or nano-pores and capillaries from which H_2_SO_4_ may be difficult to extract, even after multiple washing cycles.

**Figure 2:**
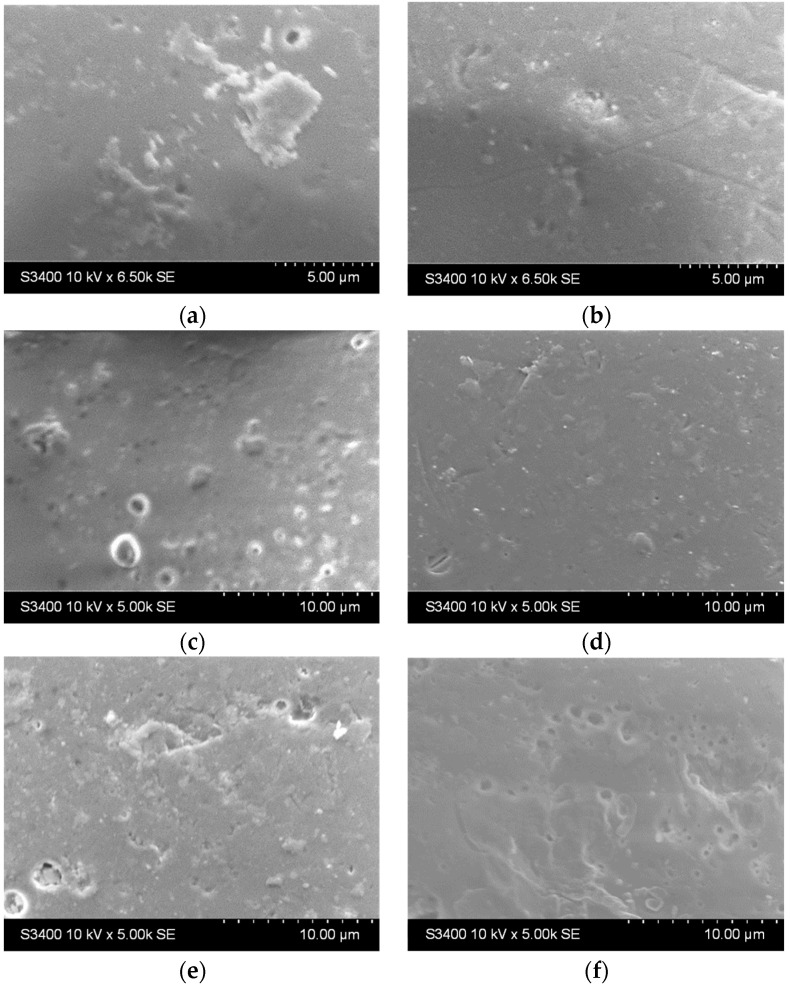
SEM images of the bead substrate (**a**) without etching and after etching with H_2_SO_4_ for (**b**) 0.5 h, (**c**) 1 h, (**d**) 2 h, (**e**) 24 h, and (**f**) 48 h.

The surface of the glass bead substrates before and after etching is presented in Figure 2. It can be noticed that the beads come with certain imperfections at the surface but are generally smooth (Figure 2a). After etching, the surface area increases (up to 2 h, Figure 2b–d). After 24 h (Figure 2e) and 48 h (Figure 2f), some craters start to form, and the surface becomes slightly damaged.

Etching in sulfuric acid could also lead to increased hydrophilicity of the substrate by the hydrolysis of Si-O bonds to Si-OH. This process could potentially lead to an improved quality of TiO_2_ thin films deposited on top through the formation of Si-O-Ti bonds. For this reason, we have performed contact angle measurements on glass slides to check the impact of H_2_SO_4_ etching on the substrate hydrophilicity. These results are included in the Supplementary Material. While the water contact angle decreases at a longer etching duration (24 and 48 h), it is not significant enough to promote better quality films deposited on top (Table S1).

To check the impact of chemical etching on the substrate composition, EDX was employed, and the results are inserted in Table 1. These confirm that longer etching durations can lead to the degradation of the surface, and traces of sulfur can be noticed. This may be due either to the infiltration of H_2_SO_4_ into the (nano-)pores or, alternatively, to the formation of metal sulfates from the reaction of sulfuric acid and metal ion impurities from the glass substrate (Na, Mg, Ca, Al).

**Figure 3:**
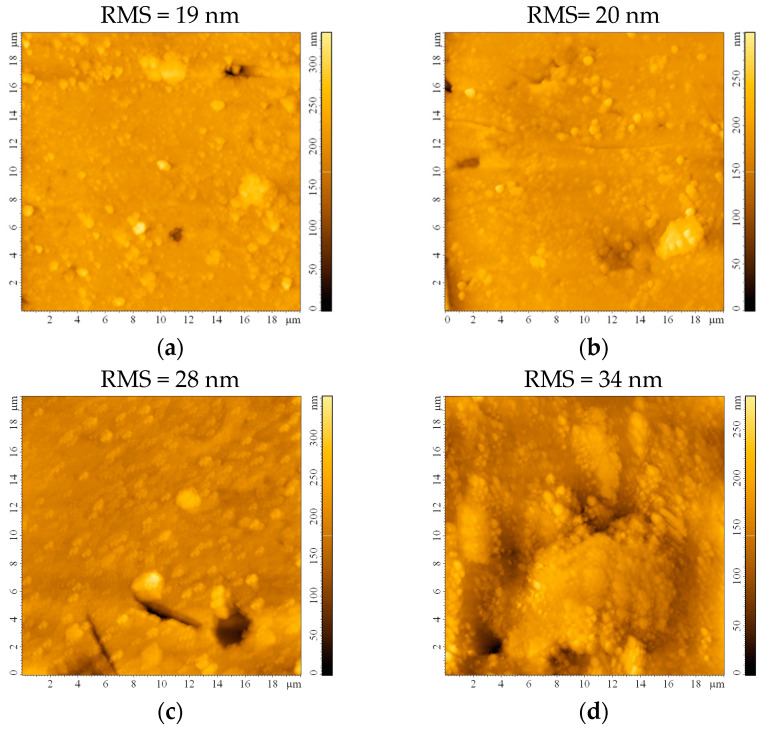

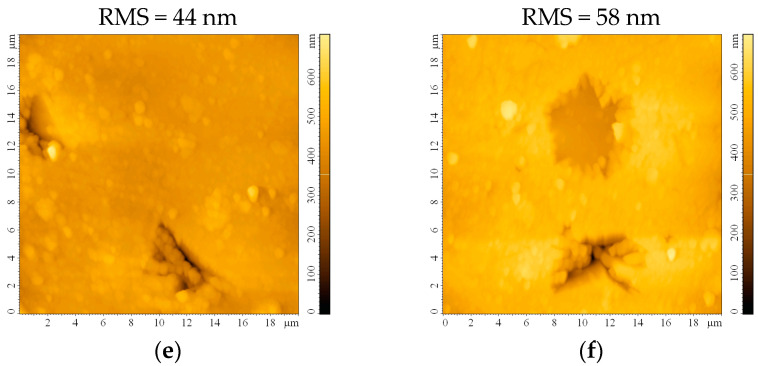
AFM images of the bead substrate (**a**) without etching or after etching with H_2_SO_4_ for (**b**) 0.5 h, (**c**) 1 h, (**d**) 2 h, (**e**) 24 h, and (**f**) 48 h.

The impact of the etching process on the surface roughness was investigated following AFM measurements on the bead substrates. The surface appears smooth, with a root mean square roughness (RMS) of 19 nm before etching (Figure 3a). This roughness maintains almost constant after 0.5 h (Figure 3b) and starts slowly increasing after 1 h (Figure 3c) and 2 h (Figure 3d) of etching. Although the RMS continues to increase to 44 nm or even 58 nm after 24 h or 48 h, respectively, the formation of small crevices/cracks on the substrate that may occur prevents these substrates from being ideal for thin film deposition. It may also be expected that sulfuric acid traces will remain on the substrate, infiltrated in the imperfections created after 24 and 48 h of etching, which would be detrimental to the thin film properties.

Based on these results, the optimum substrate is considered the one that was etched in sulfuric acid for 2 h. This was further used for the deposition of the TiO_2_ thin films using diluted or undiluted sols with one or two deposition layers. The film thickness was thus varied in order to identify the most efficient and stable photocatalyst for methylene blue degradation.

### 3.2. Influence of the Dilution of the Sol on the TiO_2_ Thin Film Properties

Two types of samples were investigated: from diluted and from undiluted sol. Their structural and morphological properties and their chemical composition were compared and correlated with their photocatalytic efficiency and stability.

**Figure 4:**
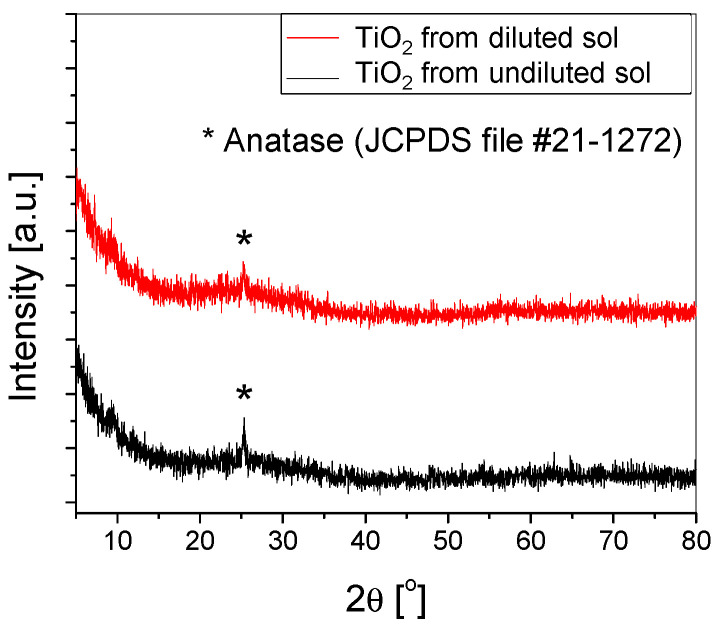
XRD of the TiO_2_ thin films deposited using (un)diluted sol on substrates etched with H_2_SO_4_ for 2 h.

The crystallinity of both types of samples was calculated based on the X-ray diffractograms (Figure 4). The value obtained from the undiluted sol was slightly higher (57%) compared to 54% for those obtained from the diluted sol. Both samples exhibit similar structural properties, with the only observable peak attributed to anatase (101).

**Figure 5:**
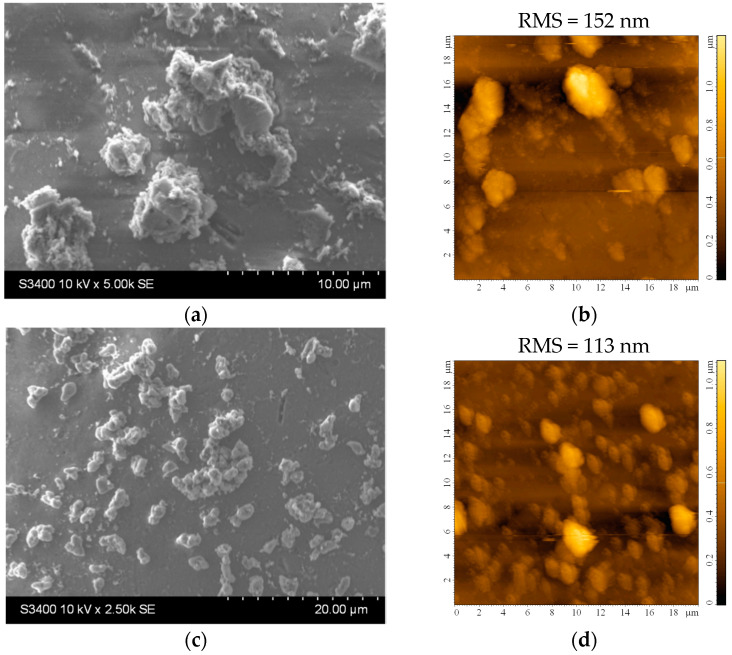
(**a**,**c**) SEM and (**b**,**d**) AFM images of the TiO_2_ thin film deposited on the bead substrate etched for 2 h in H_2_SO_4_ from (**a**,**b**) undiluted and (**c**,**d**) diluted sol.

Figure 5 shows the morphology of the TiO_2_ film covering the substrate. There are aggregates of various sizes and shapes that increase the surface area of the films. The average roughness of the films is quite large, about 100 to 150 nm for slightly larger investigated areas (20 × 20 μm^2^), as seen in Figure 5b,d. The higher roughness may be an advantage in the photocatalytic process, as it promotes increased pollutant adsorption on the photocatalytic surface. However, the stability of these aggregates in water must be confirmed to ensure they do not detach from the film surface. From the EDX analysis, it can be established that the film thickness is not uniform, and some areas of the substrate are covered with a very thin layer of TiO_2_. The amount of Ti in these areas is very low (or even undetectable), so the amount of Si and other elements (from the substrate) increases, values that can most likely be attributed to the small thickness of the film at the analyzed point.

**Figure 6:**
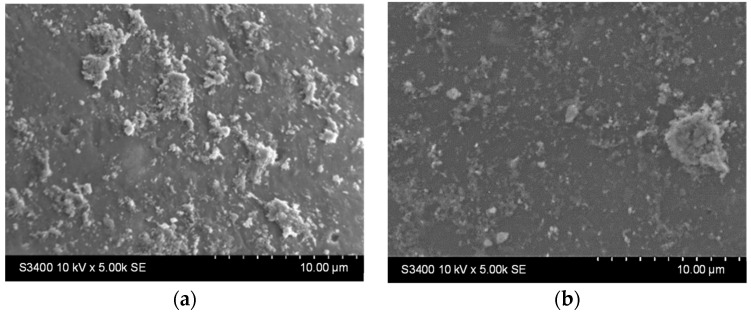

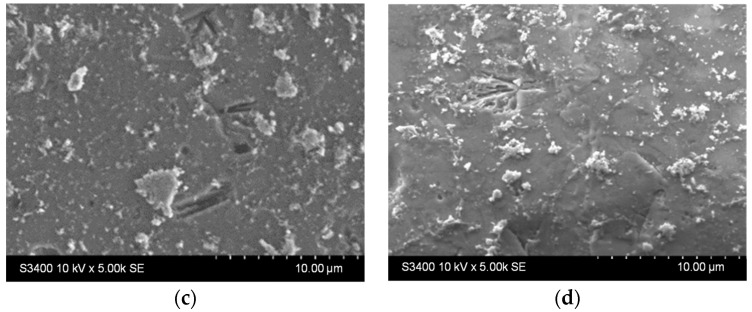
SEM images of the TiO_2_ thin film (**a**,**c**) after 9 h and (**b**,**d**) 18 h immersion in water for the samples deposited from undiluted (**a**,**b**) and diluted (**c**,**d**) sols.

Stability tests were performed by immersing the samples in distilled water (1 g photocatalytic beads in 20 mL water, under orbital stirring) for 9 h. Surface morphological changes were investigated using SEM analysis (Figure 6a,c), and then the samples were immersed for another 9 h under identical test conditions. The surface changes were investigated once more using SEM (Figure 6b,d).

It can be noticed that there are fewer larger aggregates on the surface before the stability experiments (Figure 5, Table 2). In contrast, the surface of the thin films appears to be covered with smaller, better-dispersed particles that are quite adherent, as they can be observed even after 18 h of testing (Figure 6b,d). Larger aggregates may be removed or even broken down into smaller aggregates during the first stages of immersion (during the first 9 h of testing). The slight decrease in the Ti at% concentration in the films can also be the result of the detachment of some of the larger TiO_2_ aggregates from the surface of the films, which is more pronounced in the first 9 h of testing (as also shown in Figure S1). The increase in the concentration of substrate elements (Si, Na, Mg, etc.), which can be noticed in Table 3 (correlated to the images shown in Figure S1), can explain the slight decrease in the thickness of the TiO_2_ film due to these reorganizations in the aggregates at the surface. The decrease of the Ti content after 9 and 18 h is more significant for the sample obtained using the diluted sol. This suggests that aggregate migration is more significant in this case, and therefore, the thin film is less stable in an aqueous medium. The presence of sulfur in the samples before photocatalysis is probably due to the etching treatment with H_2_SO_4_. The amount of this element increases at the surface of the samples after photocatalysis due to the methylene blue or its oxidation products (containing S) that remain adsorbed on the film surface at the end of the photocatalytic test.

**Figure 7:**
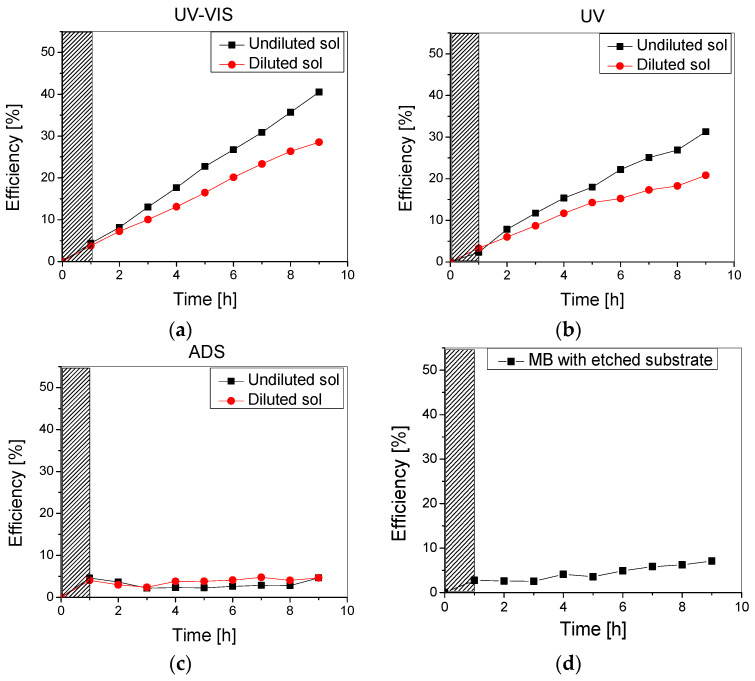
Photocatalytic efficiency of the TiO_2_ thin film obtained using undiluted and diluted sol under (**a**) UV-Vis, (**b**) UV, and (**c**) no irradiation, as well as (**d**) Methylene blue photolysis with an uncoated substrate.

Photocatalytic experiments were run using the 10 ppm methylene blue aqueous solution under UV-Vis, UV, and no irradiation (in the dark) to check the thin films’ photodegradation and adsorption efficiency. The results are presented in Figure 7.

As can be noticed in Figure 7, the samples obtained using undiluted sol had improved photocatalytic efficiency over the testing period compared to the samples using the diluted sol. This could be due to the higher amount of TiO_2_ in the film, but also following the improved stability of these films, as previously discussed.

A thicker film is preferable for enhanced photocatalytic activity since it contains more photocatalytic mass that may absorb more radiant energy. However, in the photocatalytic film, the transport of reagents relies on diffusion, which may become a limiting process if the film is relatively thick and the diffusivity is low. Moreover, the thickness of the film plays a role in determining the distribution of radiant energy intensity within it. The availability of photons at a local level, necessary for activating the reaction, depends on the depth they need to penetrate. As a result, when the optical path is relatively long, the intensity of photons can be reduced, and the reaction does not take place inside the whole film efficiently [27]. However, photocatalysis is fundamentally a surface process, and the surface morphology of the two samples plays an important role. Higher roughness means higher surface area, leading to improved photocatalytic efficiency.

Moreover, the slight differences between the photocatalytic efficiency recorded on the samples under UV-Vis (Figure 7a) and UV (Figure 7b) can be correlated to the dye-sensitization of the metal oxide thin film, as TiO_2_ is only active under UV irradiation. Finally, it is worth mentioning that while the adsorption of methylene blue on the TiO_2_ surface represents the first step in photocatalysis, it has a less significant contribution to pollutant removal as an individual process. The adsorption on both samples was almost constant over the testing period (9 h) at 3–5% (Figure 6c).

Photodegradation experiments using the methylene blue solution, with no addition of (photocatalytic) beads, were also performed in order to establish the amount of MB which is degraded through irradiation. After 1 h in the dark and 8 h of UV-Vis irradiation, following the same experimental conditions as those previously mentioned, an efficiency of 5.5% was recorded. Therefore, the difference up to 30% (for the sample obtained using the diluted sol) and up to 40% (for the sample obtained using the undiluted sol) can be mainly attributed to photodegradation and only partly to the adsorption of MB by the TiO_2_-covered beads. Moreover, during photolysis tests performed with the etched substrate (1 g) but uncoated with TiO_2_ thin film (using the same conditions as described above), a degradation efficiency of 7% was reached (Figure 7d). This increase from 5.5% to 7% was most likely due to additional MB adsorption on the glass beads substrate.

This confirms that both samples are promising photocatalysts that could potentially lead to methylene blue mineralization under solar radiation (with higher irradiance of 1000 W/m^2^). Thus, it is recommended that no dilution of the sol should be applied when aiming at depositing uniform, photoactive, and stable TiO_2_ photocatalyst on bead substrates.

### 3.3. Influence of the Number of Deposition Layers on the TiO_2_ Thin Film Properties

Another path to modify the film thickness is to sequentially deposit multiple layers. Therefore, a second TiO_2_ layer was further deposited on the glass beads previously coated with TiO_2_ using undiluted sol (previously determined as optimal).

**Figure 8:**
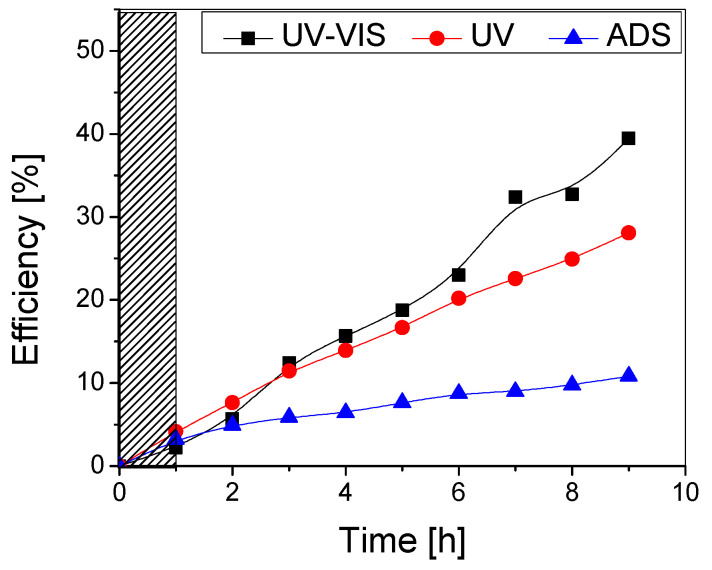
Photocatalytic (UV-VIS and UV) and adsorption (ADS) efficiency of the TiO_2_ thin film with two layers obtained from undiluted sol.

The photocatalytic performance on methylene blue removal under UV-Vis, UV, and no irradiation was investigated (Figure 8) and compared to the TiO_2_ sample with one layer (Figure 7).

It can be noticed that the addition of a second TiO_2_ layer does not significantly improve the photocatalytic performance of the samples, as the overall maximum efficiencies after the testing period (9 h) are very similar (~40% for both samples under UV-Vis and ~28% under UV irradiation). Although the double-layered sample has a higher adsorption efficiency (6% compared to 3% for the single-layered one), this did not improve the photocatalytic efficiency. Also, it is likely that some aggregates containing the adsorbed pollutant are removed from the photocatalytic surface during the testing period. In both cases, there was no flattening of the curve even after higher testing durations, which indicates that the photocatalyst surface is not affected by clogging with the pollutant or degradation by-products and may potentially lead to mineralization if (a) the process duration is extended, while maintaining the same irradiance or (b) the irradiance is increased (natural solar radiation), thus decreasing the required process duration.

To test the stability of the samples with one or two layers, their morphology and chemical composition were evaluated before and after photocatalysis. The highest impact on the samples was considered under maximum irradiance; therefore, the samples were investigated before and after testing under UV-Vis irradiation.

**Figure 9:**
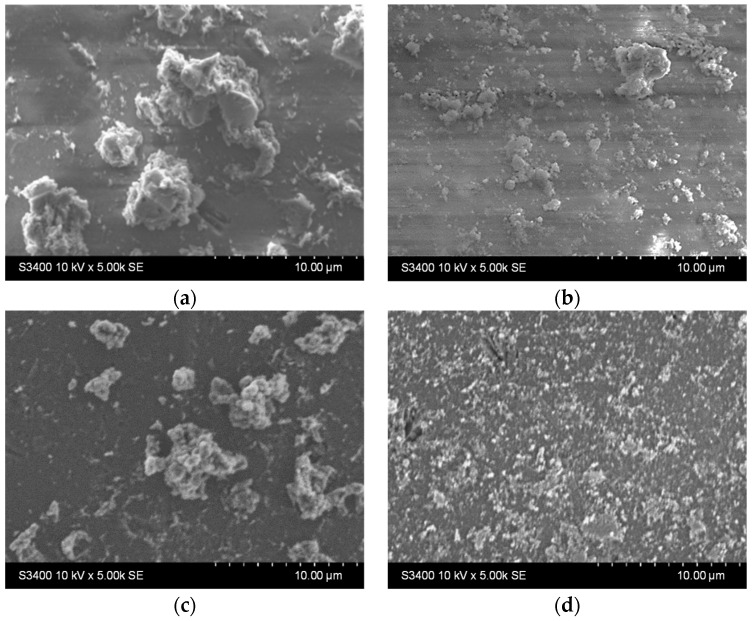
SEM images of the TiO_2_ thin films with (**a**,**b**) one and (**c**,**d**) two layers (**a**,**c**) before and (**b**,**d**) after photocatalysis.

The results presented in Figure 9 show that both samples host surface reorganization processes as the larger aggregates stacked before photocatalysis (Figure 9a,c) detach from the surface and/or are broken down into smaller aggregates after photocatalysis (Figure 9b,d). This behavior is similar to the one noticed during the stability experiments on the previously discussed films in water. Moreover, there were not any areas where film washout could be detected, either for the single-layer or for the two-layered sample.

The chemical composition at the sample surface was evaluated through EDX mapping, and the results are inserted in Table 4. The higher amount of Ti in the double-layered sample compared to the single-layered one can be due to the increased film thickness. This matches the lower substrate (Si and Na, Mg, Ca, and Al) elements contained in the double-layered film. Before photocatalysis, sulfur presence in the single-layered sample could be attributed to traces left over from the substrate etching step. After photocatalysis, sulfur content increases which could be attributed to the presence of some photodegradation by-products that were not desorbed from the photocatalyst surface. Also, after photocatalysis, the decrease in the Ti amount may be correlated with the decrease in the film thickness as a result of thin film reorganization at the surface and the partial transfer of the TiO_2_ aggregates into the solution (which is also supported by the images in Figure S2). The SEM images of the films presented before and after the photocatalytic tests (Figure 9 and Figure S2) give an indication of the entire film surface on the beaded substrate, as the process of aggregates transfer could be observed all over the surface of these samples.

The differences between the single- and double-layered TiO_2_ samples from a morphological, chemical, and photocatalytic point of view were not significant enough to recommend a multilayered structure. The increase in cost that the double layer involves offsets any slight improvement that the second layer has on the overall film properties that were investigated.

**Figure 10:**
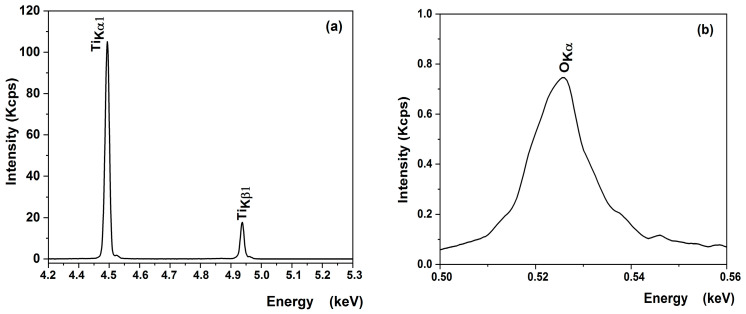
XRF spectra of (**a**) titanium and (**b**) oxygen elements of the optimized TiO_2_ thin film on bead surface.

XRF measurements were performed to verify the chemical composition of the most promising sample obtained on a substrate etched for 2 h in sulfuric acid from an undiluted sol with a single-layer deposition (Table 5). The XRF spectra of titanium and oxygen elements of the optimized TiO_2_ thin film on the bead surface illustrated in Figure 10 reveal the spectral lines of Ti_Kα1_ (4.50 eV), Ti_K*β*1_ (4.92 eV) (Figure 9a), and O _Kα_ (0.52 eV) (Figure 10b). As shown in Table 5, the experimental results are in good agreement with the theoretical ones for the film, confirming that a stoichiometric TiO_2_ was deposited.

**Figure 11:**
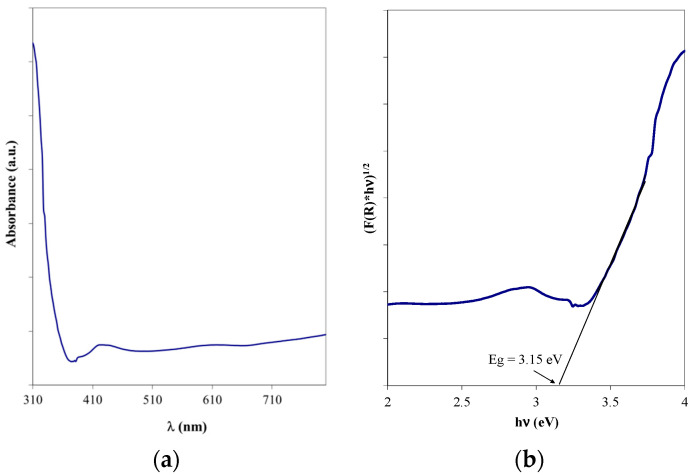
(**a**) UV-Vis spectrum and (**b**) Tauc representation for the optimized TiO_2_ thin film on bead surface.

The indirect bandgap energy, characteristic of anatase [28], was evaluated starting from the UV-Vis absorption spectrum (Figure 11a) and applying the Tauc method (Figure 11b). The obtained value was 3.15 eV and matches well with the reference literature values [29,30]; the slight differences may be the result of the surface agglomerations.

Contact angle measurements on the thin films deposited on glass beads could not be made due to the small diameter of the substrate, which offers only a curved surface for baseline determination. The wetting properties were determined for a corresponding TiO_2_ thin film obtained from undiluted sol, with one layer on 2 h etched planar glass substrate. The contact angle was ~15°, decreasing to 9.5° after UV conditioning for 9 h, as previously reported in the literature [31]. However, the differences between the chemical composition and morphology of the two substrate types do not guarantee that the values obtained on the films deposited on the planar surface would match those of the films deposited on the bead surface. The morphology and the chemical composition (or even crystallinity) of the two substrates can lead to different film growth mechanisms, thus changing their morphology and their chemical composition and, therefore, their wetting properties.

## 4. Conclusions

The study investigated the influence of various parameters on photocatalytic TiO_2_ thin films deposited on glass beads applied in the degradation of methylene blue from aqueous solutions, focusing on substrate etching duration, sol dilution, and the number of deposited layers.

The substrate etching duration in H_2_SO_4_ was adjusted to enhance surface area and nucleation sites. Longer etching time led to crater formation after 24 and 48 h, as observed in the SEM images. Chemical composition analysis using EDX confirmed sulfur traces and other elements, likely caused by H_2_SO_4_ infiltration into the pores or reactions with metal ion impurities from the glass substrate. Longer etching durations increased surface roughness (58 nm after 48 h) but hindered thin film ideal deposition due to crevices and cracks on the substrate. Residual sulfuric acid traces after etching could negatively impact thin film properties. Based on these findings, the optimum etching duration was determined to be 2 h.

This substrate was used for the deposition of TiO_2_ thin films using diluted or undiluted sols, with variations in the number of deposition layers. XRD analysis indicated similar structural properties for the thin films from undiluted and diluted sols. SEM and AFM images showed varied aggregates on the film surface, potentially enhancing photocatalytic efficiency through increased pollutant adsorption. However, the stability of these aggregates in water needed to be confirmed to ensure they did not detach from the film surface. Chemical composition analysis indicated a non-uniform film thickness, with some areas having a very thin layer of TiO_2_. Stability tests in distilled water outlined the removal or breakdown of larger aggregates into smaller ones, as shown by SEM images, particularly in the diluted sol samples, indicating reduced stability in an aqueous environment.

Photocatalysis is an advanced oxidation process that can remove trace concentration (ppm or ppb) of organic pollutants and (ideally) using solar or UV-Vis irradiation. This makes it an excellent option for advanced wastewater treatment. Photocatalytic experiments with methylene blue solution revealed that undiluted sol samples exhibited superior photocatalytic efficiency compared to diluted sol samples under various irradiation conditions. Thicker films generally exhibited enhanced photocatalytic activity due to increased photocatalytic mass, but diffusion limitations could occur in relatively thick films with low diffusivity. Higher roughness and higher surface area also supported an improved photocatalytic efficiency. On the other hand, titanium dioxide powder has a high specific surface area, thus allowing good pollutant adsorption. However, using powders as photocatalyst raises challenges related to their full recovery for reuse and limiting their dispersion in the environment. Although thin films have a smaller specific surface area than powders, they are easier to regenerate and reuse.

In conclusion, the study provides valuable insights into the impact of etching duration and sol dilution on the properties and photocatalytic performance of TiO_2_ thin films on glass beads. The findings can guide the optimization of these parameters for more efficient photocatalysts in applications such as pollutant degradation.

## Figures and Tables

**Table 1:** Chemical composition of the bead substrates according to the etching duration in H_2_SO_4_.

Etching Time	Si	O	S	Other Elements (Na, Mg, Ca, Al)
0 h	18.57	69.00	-	12.43
0.5 h	18.09	70.94	-	10.97
1 h	17.70	71.60	-	10.70
2 h	17.36	71.22	-	11.42
24 h	16.95	71.15	0.01	11.89
48 h	17.38	66.83	0.02	15.77

**Table 2:** Chemical composition of the TiO_2_ thin film obtained from undiluted and diluted sols.

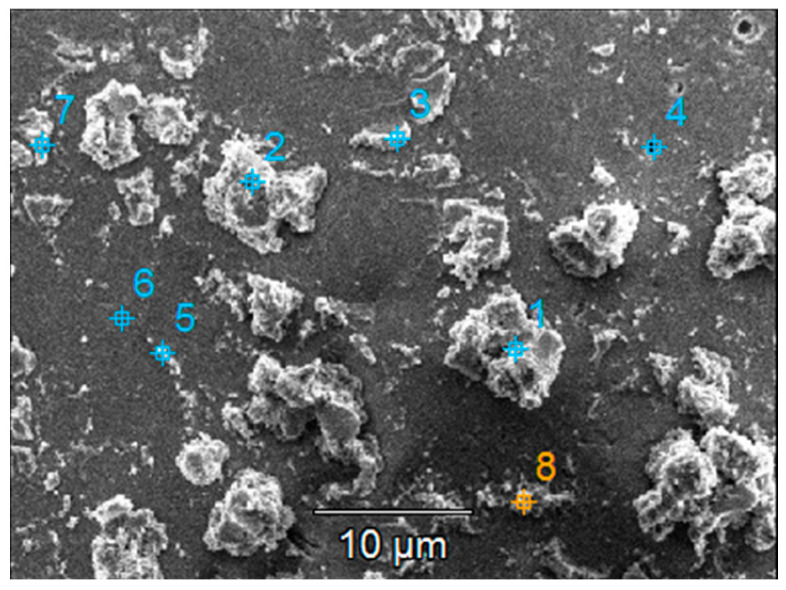		**Sample**	**Si**	**O**	**Ti**	**S**	**Other Elements** **(Ca, Mg, Na)**
	Undiluted sol	Pt1	-	84.36	15.64	-	-
		Pt2	1.36	72.11	26.53	0.00	-
		Pt3	22.44	68.76	-	0.00	8.81
		Pt4	19.40	70.25	-	0.15	10.21
		Pt5	4.56	75.16	6.48	0.59	12.85
		Pt6	11.49	75.56	-	0.25	12.70
		Pt7	12.98	78.15	0.33	0.10	8.50
		Pt8	20.32	70.07	2.15	-	7.47
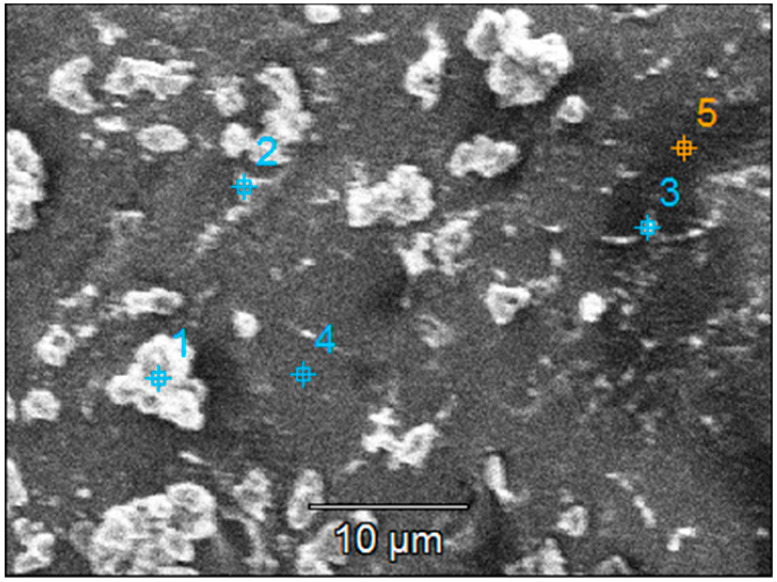	Diluted sol	Pt1	17.85	66.72	15.36	0.08	-
		Pt2	5.82	70.07	13.37	-	10.74
		Pt3	20.50	67.90	3.35	-	8.25
		Pt4	16.45	71.59	0.41	-	11.55
		Pt5	14.28	77.27	-	-	8.45

**Table 3:** Chemical composition (at. %) of the surface of the TiO_2_ layers after 9 h and 18 h of stability testing for the samples obtained using undiluted and diluted sols.

Sol Type	Stability Testing Time	Si	O	Ti	S	Other Elements (Na, Mg, Ca, Al)
Undiluted	initial	13.99	74.93	1.72	0.06	9.30
	9 h	17.02	71.48	1.43	0.06	9.99
	18 h	16.73	70.37	1.55	0.11	11.22
Diluted	initial	16.00	72.01	1.46	0.02	10.51
	9 h	16.57	71.03	1.03	0.00	11.36
	18 h	15.96	75.32	0.67	0.00	11.05

**Table 4:** Chemical composition (at. %) of the surface of the TiO_2_ thin films with one or two layers before and after UV-Vis photocatalysis (PC) of methylene blue.

No. of Layers	Stability Testing Time	Si	O	Ti	S	Other Elements (Na, Mg, Ca, Al)
1	Before PC	16.84	71.69	1.20	0.02	10.25
	After PC	14.16	74.68	0.73	0.12	10.31
2	Before PC	13.12	73.72	3.90	-	9.26
	After PC	17.38	70.85	1.71	0.12	9.95

**Table 5:** Chemical composition determined by XRF for the optimized TiO_2_ thin film on bead surface.

Chemical Element	Theoretical Chemical Composition (wt.%)	Experimental Chemical Composition (wt.%)	Absolute Error (wt.%)
Ti	59.93	60.32	+0.39
O	40.07	39.67	+0.39

## Data Availability

The data presented in this study are available on request from the corresponding author.

## Supplementary Materials

The following supporting information can be downloaded at: https://www.mdpi.com/article/10.3390/ma16144899/s1, Figure S1: SEM images of the TiO_2_ layers before (a, b) and after 9 h (c, d) and 18 h (e, f) of stability testing for the samples obtained using undiluted (a, c, e) and diluted sols (b, d, f), matching the points in Table 3; Figure S2: SEM images of the TiO_2_ thin films with one or two layers, before and after UV-Vis photocatalysis (PC) of methylene blue, matching the points in Table 4; Table S1: Water contact angle on flat microscopic glass slides.

## References

[1] Mannina G., Gulhan H., Ni B.-J. (2022). Water reuse from wastewater treatment: The transition towards circular economy in the water sector. Bioresour. Technol..

[2] Cagno E., Garrone P., Negri M., Rizzuni A. (2022). Adoption of water reuse technologies: An assessment under different regulatory and operational scenarios. J. Environ. Manag..

[3] Wang H., Li X., Zhao X., Li C., Song X., Zhang P., Huo P., Li X. (2022). A review on heterogeneous photocatalysis for environmental remediation: From semiconductors to modification strategies. Chin. J. Catal..

[4] Malato S., Maldonado M.I., Fernández-Ibáñez P., Oller I., Polo I., Sánchez-Moreno R. (2016). Decontamination and disinfection of water by solar photocatalysis: The pilot plants of the Plataforma Solar de Almeria. Mater. Sci. Semicond. Process..

[5] Wetchakun K., Wetchakun N., Sakulsermsuk S. (2019). An overview of solar/visible light-driven heterogeneous photocatalysis for water purification: TiO_2_- and ZnO-based photocatalysts used in suspension photoreactors. J. Ind. Eng. Chem..

[6] Zhang S., Zhang J., Sun J., Tang Z. (2020). Capillary microphotoreactor packed with TiO_2_-coated glass beads: An efficient tool for photocatalytic reaction. Chem. Eng. Process. Process Intensif..

[7] Wang L., Fei X., Zhang L., Yu J., Cheng B., Ma Y. (2022). Solar fuel generation over nature-inspired recyclable TiO_2_/g-C_3_N_4_ S-scheme hierarchical thin-film photocatalyst. J. Mater. Sci. Technol..

[8] Dell’Edera M., Lo Porto C., De Pasquale I., Petronella F., Curri M.L., Agostiano A., Comparelli R. (2021). Photocatalytic TiO_2_-based coatings for environmental applications. Catal. Today.

[9] Xing Z., Zhang J., Cui J., Yin J., Zhao T., Kuang J., Xiu Z., Wan N., Zhou W. (2018). Recent advances in floating TiO_2_-based photocatalysts for environmental application. Appl. Catal. B Environ..

[10] Shifu C., Gengyu C. (2005). Photocatalytic degradation of organophosphorus pesticides using floating photocatalyst TiO_2_·SiO_2_/beads by sunlight. Sol. Energy.

[11] Shen C., Wang Y.J., Xu J.H., Luo G.S. (2012). Facile synthesis and photocatalytic properties of TiO_2_ nanoparticles supported on porous glass beads. Chem. Eng. J..

[12] Ha J.-W., Do Y.-W., Park J.-H., Han C.-H. (2009). Preparation and photocatalytic performance of nano-TiO_2_-coated beads for methylene blue decomposition. J. Ind. Eng. Chem..

[13] Balakrishnan A., Appunni S., Gopalram K. (2020). Immobilized TiO_2_/chitosan beads for photocatalytic degradation of 2,4-dichlorophenoxyacetic acid. Int. J. Biol. Macromol..

[14] Khalilian H., Behpour M., Atouf V., Hosseini S.N. (2015). Immobilization of S, N-codoped TiO_2_ nanoparticles on glass beads for photocatalytic degradation of methyl orange by fixed bed photoreactor under visible and sunlight irradiation. Sol. Energy.

[15] Isik Z., Bilici Z., Adiguzel S.K., Yatmaz H.C., Dizge N. (2019). Entrapment of TiO_2_ and ZnO powders in alginate beads: Photocatalytic and reuse efficiencies for dye solutions and toxicity effect for DNA damage. Environ. Technol. Innov..

[16] Hui J., Pestana C.J., Caux M., Gunaratne H.Q.N., Edwards C., Robertson P.K.J., Lawton L.A., Irvine J.T.S. (2021). Graphitic-C_3_N_4_ coated floating glass beads for photocatalytic destruction of synthetic and natural organic compounds in water under UV light. J. Photochem. Photobiol. A Chem..

[17] Sakthivel S., Shankar M.V., Palanichamy M., Arabindoo B., Murugesan V. (2002). Photocatalytic decomposition of leather dye: Comparative study of TiO_2_ supported on alumina and glass beads. J. Photochem. Photobiol. A Chem..

[18] Sraw A., Kaur T., Pandey Y., Sobti A., Wanchoo R.K., Toor A.P. (2018). Fixed bed recirculation type photocatalytic reactor with TiO_2_ immobilized clay beads for the degradation of pesticide polluted water. J. Environ. Chem. Eng..

[19] Chand R., Obuchi E., Katoh K., Luitel H.N., Nakano K. (2013). Effect of transition metal doping under reducing calcination atmosphere on photocatalytic property of TiO_2_ immobilized on SiO_2_ beads. J. Environ. Sci..

[20] Miranda-García N., Maldonado M.I., Coronado J.M., Malato S. (2010). Degradation study of 15 emerging contaminants at low concentration by immobilized TiO_2_ in a pilot plant. Catal. Today.

[21] Ohtani B. (2010). Photocatalysis A to Z—What we know and what we do not know in a scientific sense. J. Photochem. Photobiol. C Photochem. Rev..

[22] Lee S.-Y., Park S.-J. (2013). TiO_2_ photocatalyst for water treatment applications. J. Ind. Eng. Chem..

[23] (2010). Fine Ceramics (Advanced Ceramics, Advanced Technical Ceramics): Determination of Photocatalytic Activity of Surfaces in an Aqueous Medium by Degradation of Methylene Blue.

[24] Mills A., Hill C., Robertson P.K.J. (2012). Overview of the current ISO tests for photocatalytic materials. J. Photochem. Photobiol. A Chem..

[25] Makuła P., Pacia M., Macyk W. (2018). How to correctly determine the band gap energy of modified semiconductor photocatalysts based on UV–Vis spectra. J. Phys. Chem. Lett..

[26] Souri D., Tahan Z.E. (2015). A new method for the determination of optical band gap and the nature of optical transitions in semiconductors. Appl. Phys. B.

[27] Camera-Roda G., Santarelli F. (2007). Optimization of the thickness of a photocatalytic film on the basis of the effectiveness factor. Catal. Today.

[28] Zhang J., Zhou P., Liu J., Yu J. (2014). New understanding of the difference of photocatalytic activity among anatase, rutile and brookite TiO_2_. Phys. Chem. Chem. Phys..

[29] Essalhi Z., Hartiti B., Lfakir A., Siadat M., Thevenin P. (2016). Optical properties of TiO_2_ thin films prepared by sol gel method. J. Mater. Environ. Sci..

[30] Möls K., Aarik L., Mändar H., Kasikov A., Niilisk A., Rammula R., Aarik J. (2019). Influence of phase composition on optical properties of TiO_2_: Dependence of refractive index and band gap on formation of TiO_2_-II phase in thin films. Opt. Mater..

[31] Hashimoto K., Irie H., Fujishima A. (2005). TiO_2_ photocatalysis: A historical overview and future prospects. Jpn. J. Appl. Phys..

