# Supplementary material for: Influence of the Deposition Parameters on the Properties of TiO2 Thin Films on Spherical Substrates
Source: Materials (Basel). 2023 Jul 8;16(14):4899. doi: 10.3390/ma16144899 (PMC10381302; doi:10.3390/ma16144899)

materials

Supplementary materials

Influence of the Deposition Parameters on the Properties of TiO_2 Thin Films on Spherical Substrates

Maria Covei [1], Cristina Bogatu [1], Silvioara Gheorghita [1], Anca Duta [1], Hermine Stroescu [2,*], Madalina Nicolescu [2], Jose Maria Calderon-Moreno [2], Irina Atkinson [2], Veronica Bratan [2] and Mariuca Gartner [2,*]

[1] Department of Product Design, Mechatronics and Environment, Transilvania University of Brasov, 29 Eroilor Bd., 500036 Brasov, Romania; maria.covei@unitbv.ro (M.C.); cristina.bogatu@untibv.ro (C.B.); silvioara.gheorghita@unitbv.ro (S.G.); a.duta@unitbv.ro (A.D.)
[2] "Ilie Murgulescu" Institute of Physical Chemistry, Romanian Academy, 202 Splaiul Independentei St., 060021 Bucharest, Romania; mnicolescu@icf.ro (M.N.); calderon@icf.ro (J.M.C.-M.); iatkinson@icf.ro (I.A.); vbratan@icf.ro (V.B.)
* Correspondence: hstroescu@icf.ro (H.S.); mgartner@icf.ro (M.G.)

Table S1. Water contact angle on flat microscopic glass slides.

Etching duration [h]	0.5	1	2	24	48
θ [°]	34.5	39.8	35.8	30.7	32.8

Figure S1. SEM images of the TiO_2 layers before (a, b) and after 9 h (c, d) and 18 h (e, f) of stability testing for the samples obtained using undiluted (a, c, e) and diluted sols (b, d, f), matching the points in Table 3

Stability testing time	Undiluted sol	Diluted sol
Initial	(a)	(b)
9 h	(c)	(d)
18 h	(e)	(f)

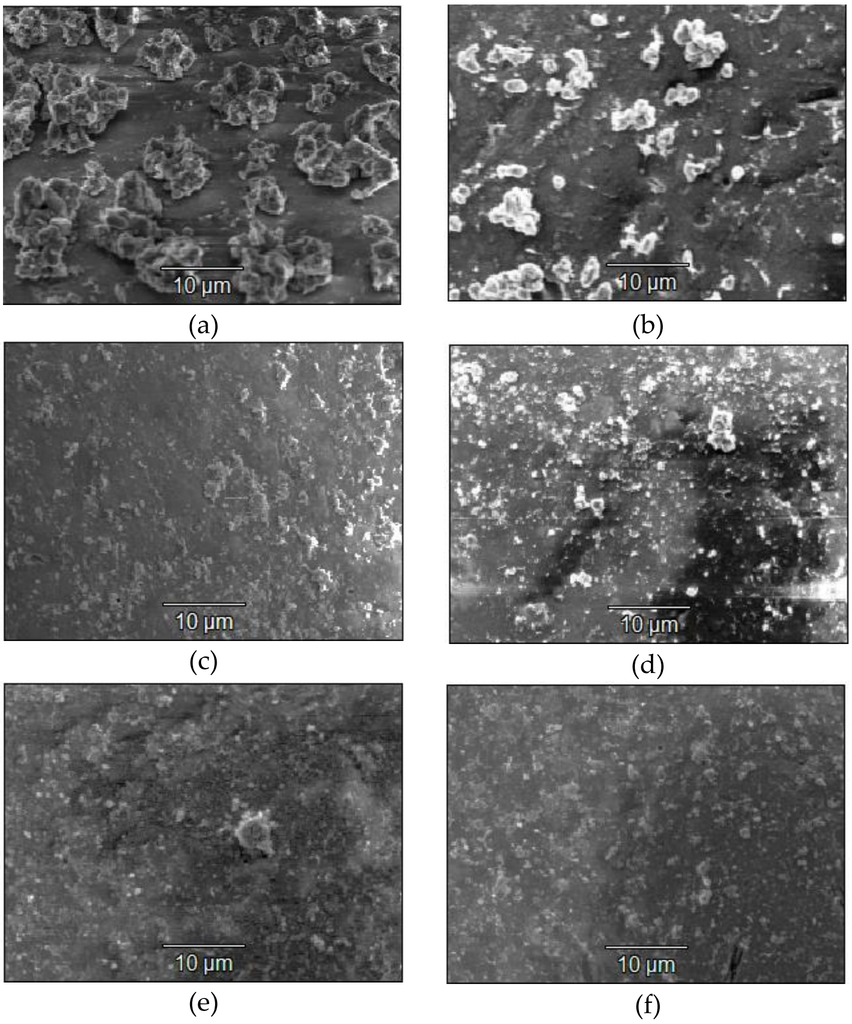

Figure S2. SEM images of the TiO_2 thin films with one or two layers, before and after UV-Vis photocatalysis (PC) of methylene blue, matching the points in Table 4.

No. of layers — Before photocatalysis — After photocatalysis

1

2

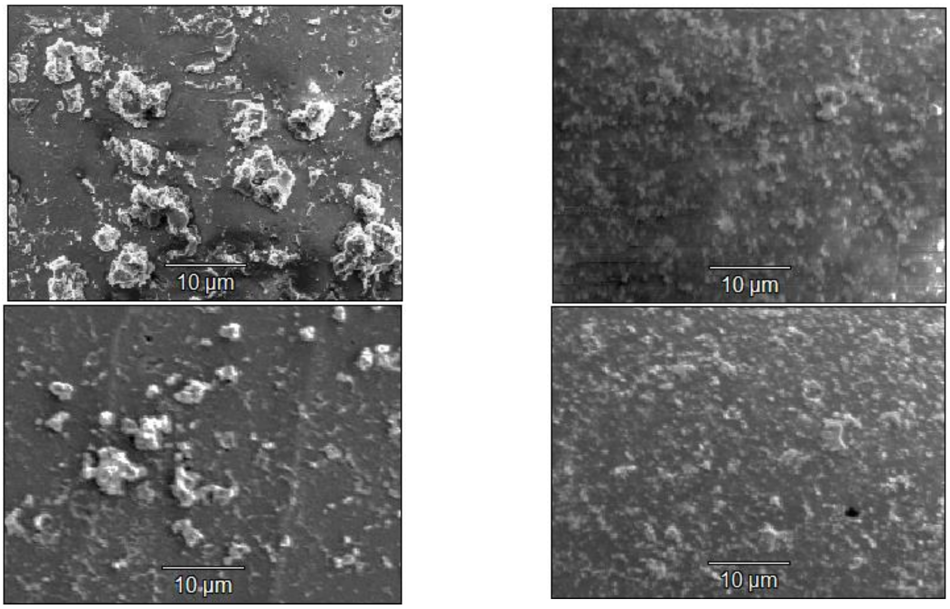

Supplement: Supplementary file 1 [file materials-16-04899-s001.zip › materials-2453765-supplementary.pdf]
